# Short-Term Fasting and Ingestion of Caloric Drinks Affect Heartbeat-Evoked Potentials and Autonomic Nervous System Activity in Males

**DOI:** 10.3389/fnins.2021.622428

**Published:** 2021-06-29

**Authors:** Vera Flasbeck, Christoph Bamberg, Martin Brüne

**Affiliations:** Division of Social Neuropsychiatry and Evolutionary Medicine, Department of Psychiatry, Psychotherapy and Preventive Medicine, LWL University Hospital, Ruhr University Bochum, Bochum, Germany

**Keywords:** interoception, heartbeat-evoked potentials, nutrition, heart rate variability, autonomic nervous system, carbohydrates, proteins

## Abstract

Central nervous systems receive and process information from the internal and external environment to maintain homeostasis. This includes interoceptive awareness of the organism’s nutritional state. Whenever food supply is required, feelings of hunger initiate the search for and the consumption of appropriate amounts of nutrients. How this is physiologically regulated in humans has been subjected to research into interoceptive awareness of body states during fasting and food consumption. However, there is no research on the distinct effects of carbohydrate or protein intake on interoception. Therefore, we aimed to investigate the impact of fasting and consumption of standardized carbohydrate and protein shakes on interoception in a repeated-measures cross-over design in a sample of 37 healthy, normal weight males. As a physiological correlate of interoception, we measured heartbeat-evoked potentials (HEPs), which are suggested to reflect the cortical representation of cardiac signals, during eight-minutes resting state EEG-recordings. After a 16-hour fasting period, the HEP amplitudes were lower over right central and parietal electrodes and increased after ingestion of the nutritional shake. Exploratory analyses indicated that the difference between fasting and satiety was more prominent at carbohydrate compared protein testing days. Correlation analyses with heart rate variability (HRV) suggested that high cardiac sympathetic activity is related to lower HEP amplitudes. Furthermore, cardiac sympathetic activity and stress indices decreased from before to after the intervention, whereas HRV increased. Together, this study shows for the first time that fasting and the intake of a nutritional shake affects cardiac measures of autonomic nervous system functioning and the neural correlates of cardiac interoception. These findings could be relevant for diets and psychosomatic disorders, including eating disorders.

## Introduction

Central nervous systems receive and process information from the internal and external environment to maintain homeostasis. This includes interoceptive awareness of the organism’s nutritional state. In general, interoception refers to the awareness of internal states, in terms of visceral sensations, including cardiovascular and gastrointestinal signals, hunger and thirst, and tactile aspects comprising pain, pressure, and temperature perception (Craig, 2002; Herbert and Pollatos, 2012; Quadt et al., 2018). Interoceptive awareness of bodily signals via the autonomic nervous system (ANS) has also been postulated as a prerequisite of emotion perception (James, 1884; Lange, 1885; Craig, 2002, 2009; Pollatos et al., 2006; Herbert and Pollatos, 2012), whereby endogenous signals serve as “somatic markers” (Damasio, 1994).

Experimentally, interoception has mainly been investigated by subjective heartbeat detection and perception tasks. Neuroimaging has shown that interoception is associated with activation of prefrontal brain regions, the somatosensory and anterior cingulate cortices, and the insula (Craig, 2002; Critchley et al., 2004; Pollatos et al., 2005, 2007b; Khalsa et al., 2009). An alternative, non-invasive method for the study of the physiology of interoception is the analysis of heartbeat-evoked potentials (HEPs), which are suggested to be a measure of neural cortical activation reflecting cardiac signal perception (Schandry et al., 1986; Schandry and Montoya, 1996). The HEPs are time-locked to the R-wave of an electrocardiogram (ECG) and can be recorded over scalp electrodes. It has reliably been shown that behaviorally assessed interoceptive awareness is associated with the magnitude of HEP amplitudes (Schandry and Weitkunat, 1990; Weitkunat and Schandry, 1990; Montoya et al., 1993; Leopold and Schandry, 2001). Thus, HEP seem to be a reliable neurophysiological measure of interoception.

Aside from interoceptive awareness of cardiac signals, previous research has examined the association of cardiac interoception with interoception of other visceral signals, as for example the perception of satiety, thirst, and gastric contractions (e.g., Whitehead and Drescher, 1980; Herbert et al., 2012b; Simmons and DeVille, 2017; Young et al., 2019). Along these lines, it has been shown that interoceptive sensitivity is related to eating behavior and body mass index (BMI). In detail, mediation analyses have shown that interoceptive sensitivity, as measured by a heartbeat-perception task, mediated the relationship between intuitive eating behavior (assessed by the intuitive eating scale) and BMI (Herbert et al., 2013). Conversely, impaired interoception has been reported for eating disorders, including bulimia nervosa, anorexia nervosa, and obesity (Pollatos et al., 2008; Klabunde et al., 2013; Herbert and Pollatos, 2014), while other studies found no differences between patients with bulimia nervosa or anorexia nervosa and healthy participants in a heartbeat detection task (Pollatos and Georgiou, 2016; Weineck et al., 2020). Similarly, whereas no difference emerged in heartbeat perception performance, Lutz and colleagues found larger HEP amplitudes in patients with anorexia nervosa compared to healthy controls (Lutz et al., 2019). Thus, the role of altered interoception in eating disorders is far from being clear.

As regards healthy individuals, Herbert et al. (2012a) reported higher interoception awareness, examined by heartbeat tracking tasks, during deliberate fasting compared to satiety (Herbert et al., 2012a). Moreover, superior interoceptive awareness during fasting was associated with subjective feelings of hunger and cardiodynamic activity (pre-ejection period, stroke volume, cardiac output, Heather index), also, heart rate variability (HRV; high frequency spectrum) was positively related to reported hunger.

Similarly, studies on HEPs demonstrated a link between HEP amplitudes and ANS functioning (Leopold and Schandry, 2001; Gray et al., 2007; MacKinnon et al., 2013). In fact, the ANS seems to be a crucial anatomical correlate of interoception, as measured by heartbeat detection or HEP (Porges, 1995). In other words, from a neuroanatomic perspective, interoceptive processing utilizes ANS network subcomponents (Craig, 2002). Accordingly, interoceptive accuracy has been linked to autonomic cardiac activity (Pollatos et al., 2007a; Herbert et al., 2012a) in ways to suggest that the ANS state affects HEP amplitudes and interoception. Conversely, one could assume that factors altering ANS function could also affect interoceptive awareness.

While most of the above mentioned studies combined the analysis of interoception measured by heartbeat detection tasks with the assessment of other internal signals such as hunger, thirst, and satiety, little is known about the associations of physiological measures, i.e., cortical representations with gastrointestinal states. Previous work has examined gastrointestinal interoception by a two-step Water Load Test, in which non-caloric water is ingested until perceived satiation (step one) and maximum subjective fullness (step two). The volume of ingested water during both steps correlated inversely with cardiac accuracy during a heartbeat perception task in healthy participants (van Dyck et al., 2016), whereas patients with bulimia nervosa and binge-eating disorder showed a delayed response to satiation compared to the control group (van Dyck et al., 2020). Another approach examines the possible association of HEP with gastrointestinal states. Schulz et al. (2015a) were the first to report increased HEP amplitudes during food deprivation (fasting for approximately 18 h) compared to normal food consumption, which consisted of a normal breakfast and a standard snack (bread with butter and cheese; Schulz et al., 2015a). No difference in ANS measures (heart rate and low frequency heart rate variability) between the food conditions emerged. Since the consumed food contained various ingredients, it is difficult to conclude which content of the breakfast might have affected interoception, since proteins und carbohydrates are digested differently. Interestingly, consuming a Western-style breakfast over a four-day period, with high intake of saturated fatty acids and added sugars reduced self-rated interoceptive sensitivity to hunger and satiety (Attuquayefio et al., 2017).

To the best of our knowledge, no study has assessed the specific impact of carbohydrates and proteins on the electrophysiological correlates of interoceptive awareness of cardiac signals and subjective feelings of hunger. Since carbohydrates and proteins are metabolized differently (i.e., uptake of glucose vs. uptake of amino acids; Franz, 1997), we expected different selective effects of those nutrients on interoception. For example glucose levels are known to affect ANS activity quickly, which is not the case for protein digestion (Landsberg and Krieger, 1989; Mizuno and Ueno, 2017). Knowledge about the impact of specific nutrients on interoception and hunger could be helpful for improving eating habits and diets. Thus, the aims of the present study were 3-fold: First, we sought to examine the impact of food deprivation and consumption on HEP in a sample of male, healthy, normal weight participants. A second goal was to assess the specific impact of protein and carbohydrate intake on HEP. Third, we aimed to analyze the association of interoception (as reflected in HEP) with ANS functioning.

## Materials and Methods

After exclusion of 2 participants, data of 37 subjects were included into the analysis. One participant was excluded because of incomplete EEG recordings and another one was excluded because he missed the second testing day. All participants were right-handed males, free of any psychological and neurological disorders, normal weight (mean BMI = 24.12, *SD* = 2.35), and non-smokers. The mean age was 25.81 years (*SD* = 3.67). Further exclusion criteria were hearing loss and substance dependence. The existence of any psychiatric disorders, including eating disorders, was excluded by the Mini-DIPS Interview (Diagnostisches Kurzinterview bei psychischen Störungen; diagnostic interview for psychological disorders; Margraf et al., 2017). Additionally, we further assessed eating disorder symptoms with the Eating Disorder Examination Questionnaire (EDE-Q; Hilbert et al., 2007). All participants gave full informed written consent. The study was approved by the Ethics Committee of the Medical Faculty of the Ruhr-University Bochum (project number 18-6378-BR) and is in accordance with the Helsinki Declaration. Unrelated behavioral results and results of another EEG-paradigm archived by this study were published previously, but are independent of the present publication (Bamberg et al., 2021a,b).

### Design and Procedure

Thirty-seven participants were recruited for the study and were asked to refrain from food, alcohol, caffeine, and caloric drinks for 16 h prior to the appointment at the study day. All study days commenced between 08:30 and 11:00 AM. At the beginning, the hearing-ability was tested and psychopathology was ruled-out by a Mini-DIPS interview. After application of the EEG-cap and placement of ECG electrodes, all subjects completed hunger and mood surveys (Abele-Brehm and Brehm, 1986) and a measurement of baseline blood-glucose was conducted. Participants marked their hunger on a visual-analog scale spanning 10 cm, ranging from “I am not hungry at all” to “I am very hungry.” The measurement of blood-glucose levels has been described elsewhere (Bamberg et al., 2021a,b). In short, 10–50 μL of blood were drawn by pricking the fingertips and the blood-glucose level was measured (in mg/dL) with a CONTOUR^®^ XT device by Bayer AG. Subsequently, a resting state EEG and parallel ECG recording followed for 8 min. Then, tones with varying loudness levels were presented to the participants for 12 min in order to analyse the so called “Loudness Dependence of Auditory Evoked Potentials” (LDAEP, results published in Bamberg et al. (2021b). For a few additional minutes, participants played the Trust Game (Bamberg et al., 2021a). Then, the participants received the drink containing either proteins or carbohydrates. After the ingestion time of 1 h, the hunger and mood surveys, the resting state EEG and ECG recording, LDAEP paradigm, and the Trust Game were repeated. At the second testing day, the same measurements were conducted except for the hearing test, diagnostic interview and questionnaires, and the Trust Game. Participants receiving carbohydrates at the first testing day received proteins at the second testing day and vice versa. The allocation of drinks to testing days was randomized. The second session of the experiment was scheduled approximately 1 week after the first (*M* = 7.06 days, *SD* = 1.6 days). As mentioned above, all participants were instructed the refrain from food and caloric drinks for 16 h. We additionally asked participants at what time they had eaten their last meal before the testing day and recorded the time of shake intake. For three participants the data regarding the time point of the last meal was incomplete. On average, the time differences between the last meal and time of appointment (carbohydrate testing day: *M* = 16:27, *SD* = 0:49; protein testing day *M* = 16:28, *SD* = 0:47) did not differ between carbohydrate and protein testing days (*Z* = −0.171, *p* = 0.864). Also, there was no difference between the time differences between the last meal and shake intake at carbohydrate and protein testing days (carbohydrate testing day: *M* = 17:45, *SD* = 1:26; protein testing day *M* = 17:29, *SD* = 0:53; *Z* = −0.684, *p* = 0.494). The participants and the experimenter were blind to the content of the drink and the results of blood glucose measurements.

### Carbohydrate and Protein Shakes

For the carbohydrate and protein shakes, 80 g of whey-protein or maltodextrin powder were solved in 400 ml of water, respectively. Both shakes were prepared in an opaque, sealed containers. The carbohydrate maltodextrin 6 was produced by Berco Arzneimittel Gottfried Herzberg GmbH (Kleve, Germany; 100 g = 380 kcal). The protein (whey-protein) powder contained a mixture of amino acids and was purchased from Fitmart GmbH & Co. KG (Elmshorn, Germany; 100 g = 374 kcal). The participants were informed that they will receive a carbohydrate shake at one day and a protein shake the other day, but they did not know the order. Although we chose the taste “neutral” for the drinks, they still differed slightly in taste. Thus, we asked the participants to wear a swimming nose clip during drinking of shakes, which reduced the identifiability of the shake. When finished, participants drank a glass of water and rinsed the mouth with Listerine (LISTERINE^®^ Cool Mint). After this procedure, they removed the nose clip.

### EEG and ECG Recording

For the resting state recording, participants sat in a reclined position in an armchair, while being instructed to avoid movements and to look at a fixation cross with a visual angle of 0.97°. The recording took place for 8 min, during which the participants were asked to alternatingly open the eyes and fixate the fixation cross and close their eyes, as previously done in studies focusing on HEP (e.g., Schulz et al., 2013, 2015a). The order of open and closed eyes was alternated between “O–C–C–O–C–O–O–C” (with “O” = “open eyes” and “C” = “closed eyes”) and “C–O–O–C–O–C–C–O.”

For recording of the data, 32 passive, non-polarizable electrodes (silver and silver-chloride), were mounted on an elastic cap (easy cap GmbH, Herrsching, Germany) according to the 10–20 system. Twenty-nine of these channels were used for EEG recordings, whereas an electrode placed at FPz was used as a ground electrode, and another one placed at FCz was used as the reference electrode. Two electrodes were used as EOG electrodes and two (bipolar) single-use silver and silver-chloride electrodes (Philips Medical Systems) were placed on each forearm close to the wrist for ECG recording. The data was amplified by a Neuvo 64 amplifier (Compumedics, Victoria, Australia) and recorded with a sampling rate of 500, 200 Hz low pass filter, using the software Curry 8 (Neuroscan Compumedics). The impedances of the electrodes were kept at 10 or below throughout the recording. From one participants, problems with ECG recording occurred, wherefore only one testing session could be analyzed for HEP and heart rate variability measures.

### Heartbeat-Evoked Potentials

EEG data was preprocessed and analyzed with the software Curry 8 (Neuroscan Compumedics). First, the recordings were down sampled to 250 Hz and segmented into *eyes open* and *eyes closed* segments. The data was further re-referenced to the mastoid electrodes and filtered (bandpass filter of 0.10 Hz with 0.2 Hz slope to 35.00 Hz with 7 Hz slope, as well as a notch filter at 50 Hz with 10 Hz width and 5 Hz slope). The Fp1 electrode was used for automatic detection of eye movements by using a threshold of ±80 μV. The marked artifacts due to eye-movements were reduced by PCA including ±200 ms before and after the artifact. Further artifacts (e.g., face muscle movements including mouth moving or swallowing, electrode cable movements) were manually marked and were omitted from the HEP analysis. The semiautomatic QRS detection, a Curry 8 feature, was used to detect R-waves in the ECG channel and to mark the R-waves in the EEG channels. The EEG data was segmented afterward according to the R-wave with −200 ms before and 800 ms after the wave. The resulting segments were averaged, corrected for the baseline (−200 ms) and HEP mean amplitudes were extracted from the timeframe 456–596 ms after the R-wave for all electrodes, as frequently done in HEP analyses (e.g., Schulz et al., 2013). We selected this timeframe, because here the electrocardiac field is suggested to be minimal, whereas it would overlap with HEP in earlier timeframes (Dirlich et al., 1997; Gray et al., 2007). The HEPs for each participant were averaged for *eyes open* and *eyes closed* conditions and for before and after proteins and carbohydrates, respectively. For the carbohydrate testing day, 205.2 segments for *eyes open* conditions (*SD* = 36.0) and 206.2 segments (*SD* = 33.3) for *eyes closed* sections were averaged for the recording before shake intake, and 211.0 segments (*SD* = 29.0) for *eyes open* conditions and 210.8 segments (*SD* = 30.1) for *eyes closed* epochs were extracted for the post-shake HEP analysis. For the protein testing day, 211.4 segments (*SD* = 34.6) for *eyes open* and 211.4 segments (*SD* = 41.6) for *eyes closed* sections were averaged for the first measurement. After consumption of the protein shake, 206.8 segments (*SD* = 34.2) for *eyes open* and 215.9 segments (*SD* = 33.6) for *eyes closed* conditions were averaged. We selected HEP amplitudes over frontal (F3, Fz, F4), central (C3, Cz, C4), and parietal (P3, Pz, P4) electrodes for statistical analyses. These electrodes were selected analogous to previous studies focusing on frontal, central, and parietal regions (e.g., Schulz et al., 2015a). The data was tested for outliers, defined as values bigger or smaller than three standard deviations. Outlying data points were deleted, and empty spaces were filled with the mean value of all cells. Two participants with several outliers were excluded from the analysis.

### Control Analyses

For additional control analyses, the ECG signal (amplitude) was also exported and the effect of the nutritional intervention on the ECG signal was investigated. Moreover, in order to control whether fasting and intake of caloric drinks would affect any ERP signal selected, we conducted the same analyses as reported for the HEP data, but for random events. Accordingly, we picked approximately (±20 events) as many events as detected by QRS detection for each participant, that is, approximately 200 segments were exported for each condition (i.e., eyes open and closed and fasting and satiety conditions). All events were corrected for baseline, averaged, and mean amplitudes were extracted from the same timeframe. Again, outliers were removed and missing values were replaced by the mean. Five participants with several outliers were excluded from the analysis. All further statistical analyses were conducted in the same way the HEP analyses were performed.

### Heart Rate Variability

Before analysis, a notch filter (50 Hz) was applied on the raw signal and data was further analyzed with the software Kubios HRV Premium (Version 3.1, Kubios Oy, Ltd.). We analyzed the data of the mean heart rate (HR), the mean RR interval, the standard deviation of NN intervals (SDNN), the root mean square of the successive differences (RMSSD), and the low-frequency (LF)/high-frequency (HF) ratio (computed by Fast Fourier Transformation). Kubios software also provides a general stress index (SI), an index for the activation of the parasympathetic ANS (PNS index), and an index reflecting the activation of the sympathetic ANS (SNS index). The stress-Index calculation (Eq. 1) is based on the median of RR intervals (*Mo*), the height of the normalized RR interval histogram (*AMo*), as well as the difference between the longest and shortest interval between R-peaks (*MxDMn*). The square-root was taken so that the *SI* was less sensitive to extreme values (Baevskii, 2002; Brugnera et al., 2018).

(1) $$SI=\sqrt{\frac{AMo\times 100}{2Mo\times MxDMn}}$$

The PNS index was based on the mean RR, the RMSSD, and the HF power. The SNS index computation was based on the mean HR, the SI, and LF power. Both indices were computed as mean deviations from normal values (Nunan et al., 2010). Thus, PNS or SNS index values of zero would indicate that the parameters reflecting parasympathetic or sympathetic activity are equal to the normal populations’ average. Indices, which deviate from zero therefore show how many standard deviations below or above the normal populations’ average the individuals’ parameter values are. Outliers (<, >3 *SD*), were deleted, participants with more than three outlying data points were excluded from the analysis (*n* = 3). These participants were also excluded from correlation analyses.

### Statistical Analyses

All statistical analyses were performed using IBM SPSS Statistics for Windows, version 26 (IBM Corp., Armonk, NY, United States). The hypotheses tested were specified before the data were collected. Because the time differences between the last meals and the appointment time as well as shake intake time were not normally distributed, non-parametric Wilcoxon tests were used for comparisons of fasting times between the carbohydrate and protein testing days (results in section “Design and Procedure”). The self-reported hunger rating was compared between time points with nutrition conditions pooled and separated by means of dependent *t*-tests. For analyses of the HEP data, repeated measures ANOVA was calculated with the factors *time point* (before the drink vs. after the drink), *nutrition* (carbohydrate testing day or protein testing day) and *eyes* (open, closed), *frontal–parietal position* (frontal, central, parietal), and *hemisphere* (left, middle, right). Greenhouser–Geisser corrected values are reported. *Post-hoc* tests were performed by using Bonferroni corrected *t*-tests. We carried out exploratory analyses for comparisons of HEP amplitudes over C4 and P4 electrodes between the time points for the separated nutritional conditions by dependent *t*-tests. For control analyses, repeated measures ANOVA were further calculated with the factors *time point* (before the drink vs. after the drink), *nutrition* (carbohydrate testing day or protein testing day), and *eyes* (open, closed) for the ECG signal. In addition, the same ANOVA and *post-hoc* tests as mentioned above for HEP data were conducted for random events of EEG data. For comparison of HRV measures before and after the shake intake and the hunger rating, dependent *t*-tests were used. Bonferroni correction was applied for comparison of heart rate variability measures due to multiple testing (significance level for the *t*-tests was set to 0.05/8 = 0.006). Effect sizes are reported as partial η*^2^* and Cohen’s *d* for ANOVA and *t*-test comparisons, respectively. Pearson correlation coefficients were calculated for the associations of HEP amplitudes (for eyes and type of nutrition pooled) over central and parietal electrodes with HRV measures for pre and post data separately. We focused only on C4 and P4 electrodes, because the other electrodes seemed to be robust against the nutritional intervention. For HRV measures, the SNS and Stress indices were included into the correlation analyses. For further analyses, the differences between pre and post data (for eyes and type of nutrition pooled; Δ = pre–post) for SNS and Stress indices and HEP amplitudes over C4 and P4 electrodes were calculated. Afterward, correlations were calculated between Δ-HRV and Δ-HEP variables. Bonferroni corrections were applied for pre, post, and Δ correlations, resulting in a significance threshold of *p* < 0.0042 (0.05/12). We calculated additional partial correlation analyses for the hunger ratings and the blood-glucose levels and HEP data over C4 and P4, again for pre, post, and Δ-data controlling for the BMI. Bonferroni correction was applied for pre, post, and Δ correlations for hunger and blood-glucose correlations, resulting in the significance threshold of *p* < 0.0083 (0.05/6). For all initial tests, a significance level of 0.05 was chosen.

## Results

The self-reported hunger decreased significantly after consumption of the drink from 5.249 (*SD* = 1.96) to 4.496 [*SD* = 2.323; *t*(35) = 2.634, *p* = 0.012; *d* = 0.350], with no difference between the type of nutrition [carbohydrates post *M* = 4.397; *SD* = 2.528; proteins post *M* = 4.594; *SD* = 2.404; *t*(35) = −0.714, *p* = 0.480; *d* = −0.080].

### Heartbeat-Evoked Potentials

**FIGURE 1:**
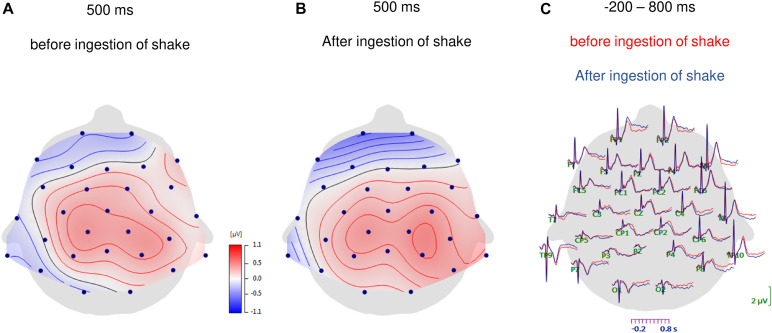
Topographic maps of HEP amplitudes at 500 ms after the R-waves for the fasting condition **(A)** and after ingestion of the shake **(B)**. In panel **(C)** the grand-average waveforms of heartbeat evoked potentials over all scalp electrodes are shown for before and after ingestion of the shake.

**FIGURE 2:**
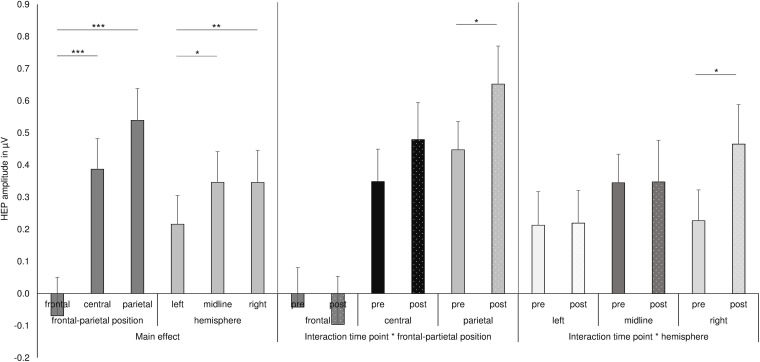
Summary of the main effects and two-way interactions of the ANOVA for the HEP data. ***p ≤ 0.001, **p < 0.01, *p < 0.05.

The analysis of HEP amplitudes by repeated-measures ANOVA with the factors *nutrition*, *time point*, *eyes*, *frontal–parietal position*, and *hemisphere* showed a main effect of *frontal–parietal position* [*F*(1.3, 41.8) = 21.34, *p* < 0.001; partial η*^2^* = 0.400] and a main effect of *hemisphere* [*F*(1.9, 60.6) = 5.94, *p* = 0.005; partial η*^2^* = 0.157]. The main effect *frontal–parietal position* indicated that HEP amplitudes were higher over central electrodes (central: *M* = 0.387, *SD* = 0.548) compared to frontal electrodes [frontal *M* = −0.069, *SD* = 0.687; central vs. frontal: *t*(32) = −5.775, *p* < 0.001, *d* = −0.734] and higher over parietal electrodes (parietal: *M* = 0.550, *SD* = 0.561) compared to frontal electrodes [frontal vs. parietal: *t*(32) = −4.777, *p* < 0.001, *d* = −0.966], whereas no difference emerged between central and parietal electrodes [central vs. parietal: *t*(33) = −1.809, *p* = 0.080, *d* = −0.242; see Figures 1A,B, 2].

As shown in Figures 1C, 2, the main effect of *hemisphere* shows that HEP amplitudes were higher at electrodes over the right hemisphere (right: *M* = 0.346, *SD* = 0.572) compared to left hemisphere [left: *M* = 0.183, *SD* = 0.489; right vs. left: *t*(32) = −3.348, *p* = 0.002, *d* = −0.306]. In addition, midline electrodes also showed higher amplitudes compared to electrodes over the left hemisphere [midline: *M* = 0.346, *SD* = 0.555; left vs. midline: *t*(33) = −2.621, *p* = 0.013, *d* = −0.243]. No difference occurred between midline and right electrodes [midline vs. right: *t*(32) = −0.305, *p* = 0.763, *d* = −0.031].

**FIGURE 3:**
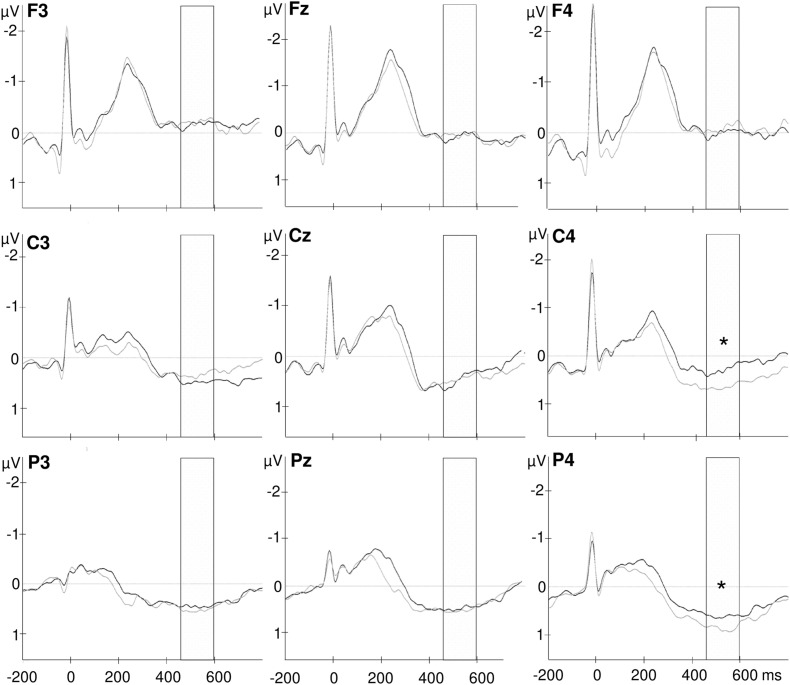
Grand-average waveforms of heartbeat evoked potentials over frontal (F3, Fz, F4), central (C3, Cz, C4), and parietal (P3, Pz, P4) electrodes. The selected time window from 456 to 596 ms after the R wave is marked in the diagram. The black lines indicate the fasting condition and gray lines represent HEPs after shake ingestion. *p < 0.05.

Most interestingly, the ANOVA further revealed an interaction of *time point* with *frontal–parietal position* [*F*(1.5, 48.5) = 4.048, *p* = 0.034; partial η*^2^* = 0.112] and an interaction of *time point* with *hemisphere* [*F*(1.7, 58.8) = 3.720, *p* = 0.036; partial η*^2^* = 0.104; see also Figure 2] and a three-way interaction of *time point* with *frontal–parietal position* and with *hemisphere* [*F*(1.8, 120.0) = 2.727, *p* = 0.036; partial η^2^ = 0.079]. *Post-hoc* tests for the interaction of *time point* with *frontal–parietal position* indicated a difference between the pre and post conditions over parietal electrodes [pre parietal: *M* = 0.448, *SD* = 0.510; post parietal: *M* = 0.652, *SD* = 0.689; *t*(33) = −2.681, *p* = 0.013, *d* = −0.338]. No differences between conditions occurred for frontal and central electrodes (pre frontal: *M* = −0.042, *SD* = 0.708; post frontal: *M* = −0.085, *SD* = 0.847; pre central: *M* = 0.349, *SD* = 0.587; post central: *M* = 0.479, *SD* = 0.670; all *p*’s > 0.05; Bonferroni correction threshold *p* = 0.05/3 = 0.017). The interaction of *time point* with *hemisphere* revealed a difference between the conditions (fasting vs. satiety) for the right hemisphere [pre right: *M* = 0.227, *SD* = 0.550; post right: *M* = 0.479, *SD* = 0.699; *t*(32) = −2.550, *p* = 0.016, *d* = −0.378]. In contrast, no differences between conditions were found for the left and midline electrodes (pre left: *M* = 0.152, *SD* = 0.504; post left: *M* = 0.219, *SD* = 0.593; pre midline *M* = 0.345, *SD* = 0.517; post midline *M* = 0.348, *SD* = 0.755; all *p*’s > 0.05; Bonferroni correction threshold *p* = 0.05/3 = 0.017). Grand-averaged waveforms for both time points and all selected electrodes are shown in Figure 3.

**FIGURE 4:**
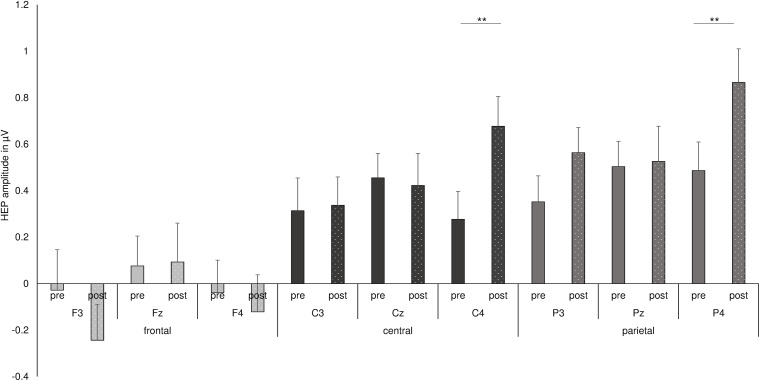
Summary of the three-way interaction of *time point* with *frontal–parietal position* and with *hemisphere*, as found by repeated measures ANOVA for HEP data. **p < 0.01.

Finally, the interaction of *time point* with *frontal–parietal position* and with *hemisphere* indicated a significant difference between the HEP amplitudes recorded before and after shake ingestion for the right central and parietal electrodes [pre C4: *M* = 0.277, *SD* = 0.697; post C4: *M* = 0.677, *SD* = 0.647; pre C4 vs. post C4: *t*(33) = −3.101, *p* = 0.004, *d* = −0.554; pre P4: *M* = 0.487, *SD* = 0.714; post P4: *M* = 0.866, *SD* = 0.842; pre P4 vs. post P4: *t*(33) = −3.017, *p* = 0.005, *d* = −0.486; see Figure 4]. The difference between conditions over P3 did not survive correction for multiple testing [pre P3: *M* = 0.352, *SD* = 0.650; post P3: *M* = 0.564, *SD* = 0.630; pre P3 vs. post P3: *t*(33) = −2.260, *p* = 0.031, *d* = −0.331]. No differences between the conditions occurred for frontal electrodes as well as for other midline and left electrodes (all *p*’s < 0.05; see also Figure 3).

Whereas no main effect of *time point*, *eyes*, or *nutrition* appeared, an interaction of *nutrition* with *frontal–parietal position* and with *hemisphere* slightly failed to reach statistical significance [*F*(3.0, 96.3) = 2.246, *p* = 0.088; partial η^2^ = 0.066]. Moreover, the interaction of *nutrition*, *time point*, *frontal–parietal position*, and *hemisphere* was not significant [*F*(3.7, 117.1) = 0.376, *p* = 0.809; partial η^2^ = 0.012]. Nevertheless, we conducted further exploratory analyses concerning the impact of nutrition and fasting on HEP amplitudes over C4 and P4 electrodes, since these electrodes have been shown to be sensitive to the *time point*, i.e., fasting and satiety states. *T*-tests between pre and post conditions for the carbohydrate and protein testing days separately showed that differences between time points were more prominent for carbohydrate testing days than for protein testing days [carbohydrates C4 pre *M* = 0.179, *SD* = 0.804; post: *M* = 0.666, *SD* = 0.881; carbohydrates C4 pre vs. post C4: *t*(33) = −2.826, *p* = 0.008, *d* = −0.578; carbohydrates P4 pre *M* = 0.383, *SD* = 1.028 post: *M* = 0.927, *SD* = 0.902; carbohydrates P4 pre vs. post C4: *t*(33) = −2.689, *p* = 0.011, *d* = −0.563]. Comparisons of time points for the protein testing day showed no statistical significant differences (all *p*’s > 0.05).

Finally, as multiple-comparisons by ANOVA are fraught with the problem of inflated Type I error (Cramer et al., 2016), we note that in the present study only the two main effects survived Bonferroni–Holm correction for controlling familywise error rate (controlling for 16 relevant main effects and interactions).

### Heart Rate Variability

The comparison of HRV measures between before and after consumption of the shakes revealed significant differences for the SNS index, SI, SDNN, and LF/HF ratio (see Table 1). The SNS and Stress indices and LF/HF ratio decreased from before to after the intervention, whereas SDNN was increased. When applying Bonferroni correction, due to multiple testing, the differences remained significant for the SNS and Stress indices. No differences in HRV between the shakes, i.e., proteins or carbohydrates occurred.

**TABLE 1:** Comparison of HRV measures before (pre) and after (post) consuming the carbohydrate and protein shakes, irrespective of the drink’s content.

HRV measure	pre *M* (*SD*)	post *M* (*SD*)	*t*	*p*	*d*
PNS index	0.2243 (1.36)	0.3994 (1.21)	–1.913	0.065	–0.333
SNS index	0.1530 (1.16)	−0.1134 (0.98)	3.022	0.005*	0.526
Stress index	9.5874 (3.14)	8.6446 (2.40)	3.126	0.004*	0.544
Mean RR (ms)	928.1665 (122.06)	938.8516 (114.75)	–1.108	0.276	–0.193
SDNN (ms)	60.3923 (20.52)	66.01 (19.67)	–2.803	0.009*	–0.488
Mean HR	65.1120 (7.38)	64.1820 (6.92)	1.354	0.185	0.239
RMSSD	47.4165 (24.81)	50.1565 (20.39)	–1.623	0.114	–0.283
LF/HF ratio (FFT)	1.9530 (1.37)	1.6299 (1.19377)	2.148	0.039*	0.374

Mean and standard deviations are shown, as well as statistic of dependent *t*-tests (*t*, *p*, *d*). Significant differences are marked with * and differences remaining significant after Bonferroni-correction (*p* = 0.05/8 = 0.006) are printed in bold (*n* ≥ 32).

### Correlations

**FIGURE 5:**
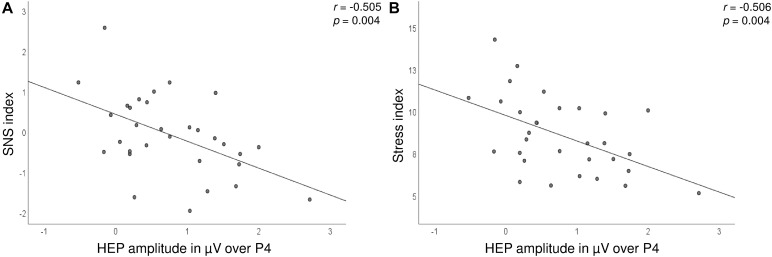
Scatterplot showing the correlations between the SNS **(A)** and Stress **(B)** indices (*y*-axis) and HEP amplitudes over the P4 electrode (*x*-axis) after the consumption of the drink.

Significant correlations between the HEP amplitudes and HRV measures were found during the satiety conditions. In detail, the Stress and SNS indices were negatively associated with HEP amplitudes over the P4 electrode, which suggest lower amplitudes during high SNS activity (Figure 5 and Table 2). The correlations for Δ pre–post data for P4 electrode as well as correlations over C4 for post data did not survive correction for multiple testing.

**TABLE 2:** Correlations between HEP amplitudes over central (C4) and parietal (P4) electrodes and HRV measures (SNS and Stress indices) before (pre) and after (post) the nutritional intervention.

	C4			P4		
	Pre	Post	Δ	Pre	Post	Δ
SNS index	−0.020 (0.915)	−0.380* (0.035)	0.332 (0.068)	−0.102 (0.585)	−0.505* (0.004)	0.360* (0.047)
Stress index	−0.004 (0.984)	−0.378* (0.036)	0.240 (0.194)	−0.061 (0.745)	−0.506* (0.004)	0.392* (0.029)

Calculations were conducted for HEP pre with HRV pre and HEP post with HRV post data. In addition, correlations were calculated for the difference Δ pre–post for the above mentioned variables. Significant correlations are marked with * (*n* ≥ 30) and correlations surviving correction for multiple testing are printed in bold (*p* < 0.0042).

### Additional and Control Analyses

In order to check whether differences in HEP amplitudes could be simply related to differences in ECG signals during the selected time window, we calculated repeated-measures ANOVA with the factors *nutrition, time point*, and *eyes* for the ECG amplitudes. Here, neither main effects of *nutrition*, *time point*, and eyes occurred, nor interactions with *time point* were found, as observed for HEP data (all *p*’s > 0.05). One significant interaction of *nutrition* with *eyes* occurred, whereas *post-hoc* test between nutritional interventions and eyes open/closed conditions failed to reach significance (all *p*’s > 0.05). For another control analysis, random ERP events were created and the analysis conducted for HEP data was repeated. Here, only a main effect of *eyes* occurred [*F*(1.0, 30.0) = 9.33, *p* = 0.005; partial η^2^ = 0.237] indicating lower amplitudes when eyes were open compared to eyes closed recordings (eyes open: *M* = −0.221, *SD* = 0.388; eyes closed: *M* = 0.030, *SD* = 0.452). In addition, an interaction of *eyes* with *frontal–parietal position* was observed [*F*(1.8, 55.5) = 7.32, *p* = 0.002; partial η^2^ = 0.196], which indicated a difference between eyes open and closed recordings only over frontal electrodes [frontal eyes open *M* = −0.393, *SD* = 0.607; frontal eyes closed *M* = 0.052, *SD* = 0.550; frontal eyes open vs. closed: *t*(30) = −3.936, *p* < 0.001, *d* = −0.769] and a differences between frontal and parietal during the eyes open condition [parietal eyes open *M* = −0.093, *SD* = 0.400; frontal eyes open vs. parietal: *t*(30) = −3.487, *p* = 0.002, *d* = −0.583]. An interaction of *nutrition* with *frontal–parietal position* further occurred [*F*(1.8, 54.8) = 4.88, *p* = 0.013; partial η^2^ = 0.140], whereas no significant effect surviving Bonferroni correction (*p* < 0.006) was found. The same applies for the interaction *time point* with *frontal–parietal position* and *hemisphere* [*F*(3.6, 108.03) = 2.88, *p* = 0.031; partial η^2^ = 0.087], where no *post-hoc* test reached significance (all *p*’s > 0.05).

Finally, we calculated correlation coefficients between HEP data and the subjective rating and blood-glucose levels. Here, only the correlation between blood-glucose level and HEP over P4 during fasting reached significance (Table 3).

**TABLE 3:** Partial correlations between HEP amplitudes over central (C4) and parietal (P4) electrodes, hunger ratings, and blood glucose levels controlling for the variable BMI.

	C4			P4		
	Pre	Post	Δ	Pre	Post	Δ
Hunger	0.075 (0.684)	0.074 (0.686)	0.344 (0.054)	0.118 (0.521)	0.035 (0.849)	−0.178 (0.329)
Blood-glucose level	−0.222 (0.223)	0.028 (0.880)	0.185 (0.312)	−0.465 (0.007)*	−0.119 (0.515)	−0.170 (0.351)

Correlation were calculation for pre, post, and for the difference Δ pre–post data. Significant correlations are marked with * (*n* = 30) and correlations surviving correction for multiple testing are printed in bold (*p* < 0.0083 for hunger ratings and blood glucose measure separately).

## Discussion

The present study aimed to characterize the effect of fasting and selective intake of carbohydrates and proteins on the electrophysiological correlate of cardiac interoception, namely HEP in a sample comprising healthy, male participants. We found higher HEP amplitudes during the satiety condition compared to the fasting condition over right central and parietal electrodes, whereas in the initial analyses, no effect of the type of nutrition occurred. This findings are in contrast to the study of Schulz et al. (2015a), who reported increased HEP amplitudes after an 18-hours fasting period compared to normal food intake (Schulz et al., 2015a). As mentioned before, there is, to the best of our knowledge, no other study that examined cortical representations of vascular-afferent signals and effects of fasting and nutritional uptake. In a behavioral study, food deprivation for 24 h has been shown to lead to increased interoceptive accuracy, as measured by heartbeat detection tasks, which was related to increased feelings of hunger (Herbert et al., 2012a). Thus, these two studies suggest that short-term fasting might increase the awareness of bodily sensations, especially visceral-afferent signals, resulting in an increased feeling of hunger, which might serve as motivational driver to induce adaptive behavior in terms of food acquisition. However, these two studies recruited exclusively female participants, whereas only male participants took part in our experiment. We aimed to avoid confounding effects of the menstrual cycle on HEP or interceptive awareness, since research has demonstrated an association between menstrual cycle and food craving and food intake (e.g., Dalvit, 1981; Hill and Heaton-Brown, 1994). Differences in heartbeat detection were further previously suggested to be associated with cardiodynamic parameters, including the stroke volume (Schandry et al., 1993), which could also contribute to differences between males and females. Finally, the proposed mechanisms could differ in males, in terms of lower interoception during fasting, which could bear the advantage that attentional resources can be allocated away from internal sources to more external sources. After food consumption, sensitivity to visceral-afferent signals may increase, via hormonal pathways (through digestion of consumed food products, followed by insulin secretion, etc.), and neural pathways signaling fullness (through stretch receptors in the gastrointestinal system). Since no interaction with type of nutrition was found initially in the present study, one could suggest that the signaling of fullness could play a pivotal role in regulating interoception. This is further supported by the correlations of low HEP amplitudes over P4 with high SNS activity after drink consumption. It is important to note that the SNS and stress indices decreased from before to after the intervention. Thus, the fasting condition seem to induce stress in terms of higher activity of the sympathetic nervous system, with food consumption decreasing the sympathetic activity. Similarly, a study by Chan and colleagues showed that fasting for 72 h was associated with decreased parasympathetic cardiac modulation (Chan et al., 2007). Moreover, 2 days of fasting seem to induce a shift toward greater sympathetic activity in overweight individuals compared to normal-weight subjects (Wijngaarden et al., 2013; Solianik and Sujeta, 2018). While feelings of satiety are mediated by vagal afferents (Guarino et al., 2017), the present study additionally suggests that interoception (i.e., amplitude of HEP) is associated with low sympathetic ANS activity in satiated individuals. In support of the association of HRV with HEP, we showed in a previous study with patients with personality disorder and healthy participants that HEP amplitudes were positively associated with PNS activity (Flasbeck et al., 2020). In contrast, Schulz et al. (2015a) did not find associations of HEP with heart rate and normalized low frequency HRV. The behavioral study of Herbert et al. did report associations of increased interoceptive accuracy and cardiodynamic activity and HRV during fasting (Herbert et al., 2012a). In a study with patients suffering from depersonalization/derealization disorder, HEP amplitudes were not related to cardiovascular measures of sympathetic nervous system activity. With the behavioral Whitehead tasks, however, negative correlations emerged with normalized low-frequency HRV and the LF/HF ratio (Schulz et al., 2015b). Another study suggested that HRV influences the afferent input to the brain, measured as HEPs, during the experience of emotion as well as during resonant breathing (MacKinnon et al., 2013). Together, these results suggest an effect of ANS functioning on interoception and HEPs, whereas the extent varies across studies and conditions. More evidentiary, previous work and our own project demonstrated that fasting and food intake affect interoception of cardiac signals and the cortical representation thereof. However, the associations of HRV and HEP may be more prominent under “normal” condition and altered under the “stressing” fasting condition.

In our study, we did not assess interoceptive accuracy, which is why the results of our study using HEPs are difficult to compare with behavioral studies. Therefore, based on our data, we cannot conclude that HEP changes reflect changes in visceral interoception. In fact, hunger rating did not correlate with HEP amplitudes. In contrast, the blood-glucose level correlated inversely with HEP amplitudes during fasting. This could suggest that subjective interoceptive accuracy and objective measures such as HEP are not that tightly correlated as predicted (Coll et al., 2021). Further parameters varying across studies is the fasting duration and the type and amount of food consumed. Moreover, even though the hunger rating decreased after the drink consumption, the participants reported being still hungry (hunger rating 4.5 on a scale from 0 to 10, where 0 was “not hungry”). Because the caloric value of the drinks was about 300 kcal, the findings invite speculations that the feeling of hunger is perhaps mainly induced by perceived satiety or reflects the subjective impression of not having eaten a “real meal.” Alternatively, the absence of the experience of taste might also lead the impression of not having eaten. This idea is supported by the fact that hunger ratings did not differ between the nutrition conditions, whereas blood glucose levels were differentially affected by the condition (Bamberg et al., 2021a). This suggests that the subjective feeling of hunger may differ from physiological states since carbohydrates selectively impact on blood glucose levels, but not HEP amplitudes in the first place. However, exploratory analyses of the impact of carbohydrates and proteins und HEP amplitudes revealed a significant difference between time points only for the carbohydrate testing day, but not for the protein testing day. This invites speculation that carbohydrate and protein metabolism affect the perception of cardiac signals in different ways, possibly via differential ANS activation (Landsberg and Krieger, 1989; Mizuno and Ueno, 2017). The mechanisms and the reliability of these findings discovered by exploratory analyses should be investigated by future research, however. It would also have been interesting to include a third condition in which subjects would receive a non-caloric drink (e.g., plain water) of the same volume, which should be tested in future studies. Together, we hypothesize that the feeling of hunger as well as HEP amplitudes were mainly modulated by the gastric distension than by hormonal pathways induced by the nutritional composition.

Indeed, the nutritional composition has been shown to selectively affect another electrophysiological marker, the LDAEP, which has been proposed to be related to the activity of the neurotransmitter serotonin in the brain, as suggested based on additional data of the present study. More detailed, the marker for central serotonergic activity was increased after food intake compared to fasting conditions, whereas this effect was more prominent for the carbohydrate consumption than after proteins (Bamberg et al., 2021b). Thus, the nutritional composition impacts on various physiological processes via hormonal pathways and metabolism of the nutrients, whereas subjective feelings of hunger and probably also electrophysiological correlates of cardiac interoception might be more affected by gastric distension. However, this interpretation is speculative since we did not assess whether fasting and satiety states might induce general differences in physiological and brain states, which is probably not specific and limited to HEP. The control analysis for random EEG events at least showed that there was no effect of fasting and nutrition conditions on random ERPs. Finally, we also note that attentional changes could have affected the HEP amplitudes. When feeling satiated, the participants might have shifted their attention, a factor which has been frequently shown to affect HEPs (Coll et al., 2021). Together, this finding could be relevant for the understanding of eating habits and diets aiming at weight reduction. The experience of hunger (or deliberate dieting) might induce an increase in SNS activity which further alters interoceptive processing. One could suggest that during states of high SNS activity, the attention could be allocated to the environment in order to gather food. After food consumption, the HEP amplitudes were increased, because of lower SNS activity and higher PNS activity. In a previous study, it has been shown that timing of meals was a key factor in regulating circadian phases of the cardiac ANS (Yoshizaki et al., 2013). And, interestingly, diet and exercise based weight loss was related to increased parasympathetic and decreased sympathetic activity (Costa et al., 2019). Whether or not these insights could be relevant for the diagnosis or treatment evaluation of eating disorders needs to be addressed in future research.

In our study, the effects observed in our HEP analyses occurred mainly over the C4 and P4 electrodes, i.e., the right central brain region. The localization of maximal HEP amplitudes over frontal and central electrodes has been reported frequently and is in accordance with the idea of the anterior cingulate cortex, insular, parietal, somatosensory, and somatomotor cortices being involved in interoception (Craig, 2002; Critchley et al., 2004; Pollatos and Schandry, 2004; Pollatos et al., 2005, 2006, 2007b; Kern et al., 2013; Park et al., 2014; Canales-Johnson et al., 2015; Babo-Rebelo et al., 2016; Azzalini et al., 2019). A higher involvement of the right hemisphere has been demonstrated previously (Leopold and Schandry, 2001; Pollatos and Schandry, 2004). Moreover, it has been suggested that the responses in the somatosensory cortices reflect contralateral signals (Hari et al., 1993). For the investigation of the involvement of deeper brain regions, for instance the anterior cingulate cortex or the insular cortex, fMRI would be a more elaborated and suitable tool. Therefore, one limiting factor of the present study is the low spatial resolution based on the chosen method for the detection of HEPs. Another limitation of our study, as mentioned before, is the inclusion of exclusively male participants, wherefore results can be interpreted to be meaningful only for males. Another limitation of the present study is the lack of a behavioral task assessing interoceptive performance, for example a heartbeat-perception task.

### Conclusion

This is the first study showing that fasting and intake of carbohydrates and proteins affect HEP amplitudes, whereas this effect was more prominent during the carbohydrate testing days, as shown by exploratory analyses. These results are restricted to a sample of male, normal weight, young participants. The electrophysiological marker was further associated with HRV measures indicating that low sympathetic activity is related to high cortical representations of cardiac signals under satiated states.

## Data Availability Statement

The data will be made available by the corresponding author upon request.

## Ethics Statement

The studies involving human participants were reviewed and approved by Ethics Committee of the Medical Faculty of the Ruhr-University Bochum. The patients/participants provided their written informed consent to participate in this study.

## Author Contributions

VF: acquisition, analysis, interpretation of data for the work, and drafting the work. CB: acquisition and revising the manuscript critically for important intellectual content. MB: interpretation of data for the work and revising the manuscript critically for important intellectual content. All authors: substantial contributions to the conception or design of the work, final approval of the version to be published, and agreement to be accountable for all aspects of the work.

## Conflict of Interest

The authors declare that the research was conducted in the absence of any commercial or financial relationships that could be construed as a potential conflict of interest.
